# Dependence of prevalence of contiguous pathways in proteins on structural complexity

**DOI:** 10.1371/journal.pone.0188616

**Published:** 2017-12-12

**Authors:** Kelly M. Thayer, Jesse C. Galganov, Avram J. Stein

**Affiliations:** 1 Department of Mathematics and Computer Science, Wesleyan University, Middletown, CT, United States of America; 2 Program in Molecular Biophysics, Wesleyan University, Middletown, CT, United States of America; 3 Department of Chemistry, Wesleyan University, Middletown, CT, United States of America; 4 Program in Bioinformatics, Wesleyan University, Middletown, CT, United States of America; 5 Department of Astronomy, Wesleyan University, Middletown, CT, United States of America; 6 Department of Earth and Environmental Sciences, Wesleyan University, Middletown, CT, United States of America; Wake Forest University, UNITED STATES

## Abstract

Allostery is a regulatory mechanism in proteins where an effector molecule binds distal from an active site to modulate its activity. Allosteric signaling may occur via a continuous path of residues linking the active and allosteric sites, which has been suggested by large conformational changes evident in crystal structures. An alternate possibility is that the signal occurs in the realm of ensemble dynamics via an energy landscape change. While the latter was first proposed on theoretical grounds, increasing evidence suggests that such a control mechanism is plausible. A major difficulty for testing the two methods is the ability to definitively determine that a residue is directly involved in allosteric signal transduction. Statistical Coupling Analysis (SCA) is a method that has been successful at predicting pathways, and experimental tests involving mutagenesis or domain substitution provide the best available evidence of signaling pathways. However, ascertaining energetic pathways which need not be contiguous is far more difficult. To date, simple estimates of the statistical significance of a pathway in a protein remain to be established. The focus of this work is to estimate such benchmarks for the statistical significance of contiguous pathways for the null model of selecting residues at random. We found that when 20% of residues in proteins are randomly selected, contiguous pathways at the 6 Å cutoff level were found with success rates of 51% in PDZ, 30% in p53, and 3% in MutS. The results suggest that the significance of pathways may have system specific factors involved. Furthermore, the possible existence of false positives for contiguous pathways implies that signaling could be occurring via alternate routes including those consistent with the energetic landscape model.

## 1. Introduction

Allosteric regulation of protein function occurs when the binding of an effector modulates the protein’s interaction with a ligand at a distal site [1,2]. While allosteric regulation has been appreciated as an empirical observation for some half a century, understanding how the signal propagates remains an active area of research. Early descriptions of the phenomenon are attributed to Monod, Wyman and Changeux (MWC model) [3–5] and to Koshland, Nemethy, and Filmer (KNF model) [6] on the basis of structural studies of cooperativity in hemoglobin. Allosteric control has since been recognized as playing a critical role in feedback mechanisms, such as in the CAP transcription factor [7–9], signal transduction cascades [10], G protein-coupled receptors [11,12], ion channels [13] and enzymes [14–18] and many other functions involving conformational changes in the dynamic structure of proteins [19]. Understanding the allosteric signaling mechanism in greater detail is not only promising for elucidating protein specific knowledge, but also in developing allosteric drugs with a rational design approach rather than an empirical screen [20–22].

Several ideas regarding allosteric signal propagation have been proposed. Conformational changes observable in crystal structures upon binding of an effector in an initial and final state suggest a mechanical view can be adopted. Some examples exhibiting large conformational changes include rotary ATP synthase [23], Ras GTPase [24], Beta adrenergic receptor [25], the ribosome [26], dihydrofolate reductase [27], and calmodulin [28,29]. This type of analysis lends itself to the idea that a signal could be propagated through a network of contiguous residues in contact with each other.

However, allosteric signaling in the absence of a conformational change is also possible. The idea was first put forth by Cooper and Dryden in 1984 on a theoretical basis [30]. The work of Hilser and coworkers [31,32] has further elaborated on the idea, arguing that changes in the energy landscape could in essence be the allosteric signal, but that the ensemble averaged structure of the protein would not necessarily be changed and therefore could escape experimental detection.

Recent studies in this vein [33–35] involved examining a variety of theoretical coarse grain models to study propagation of allosteric signals. They conclude that there is a requirement for inhomogeneous elastic density, which could be due to differences in rigid and less rigid areas of the protein. The signal propagation could occur through changes in the thermal fluctuations [35]. Hilser and coworkers explored the statistical thermodynamics of the protein ensemble with their COREX coarse grain software [32]. Allostery without a conformational change has been observed in methionine repressor on the basis of crystal structure B factor changes and NMR data [36]. The cyclic AMP receptor protein has been experimentally shown to change its affinity for cAMP mediated only through changes in dynamics [37]. Our recent studies of allosterism in MutS, a multidomain protein in which the allosteric signal is propagated over 100Å, we identified a network of contiguous residues by SCA [38]. Testing of signal disruption via MD simulations on alanine mutants indicated a disruption of essential H-bonds in the active site. However, we also found that the alanine mutants in the network but not in the contiguous pathway also exhibited a similar disruptive effect, which could be evidence for the energetic landscape model coexisting with a pathway mode of signal transmission. Interestingly, ay similar observation of multiple networks has emerged for the PDZ domain by the SCA method described below. This protein has had a network of coevolving residues identified by SCA analysis [39]. However, an NMR study [40] revealed an allosteric regulatory role for its third alpha helix not found in other homologs. Energy fluctuation calculations also on PDZ [41] identified two independent networks. The result could indicate coexistence of two independent networks operating by different mechanisms.

The identification of networks of residues in proteins has been carried out by several methodologies. The package PSN-Ensemble takes snapshots from MD, NMR, or several crystal structures to represent an ensemble of structures and considering cross-correlations to elucidate allosteric pathways as a cooperative network [42]. Bahar and coworkers have developed elastic rod networks, and have recently reported a comprehensive database of their results on most all structures in the PDB [43,44]. An alternate means of identifying pathways has been presented with the algorithm in allopathfinder that predicts pathways of residues on the basis of distance constraints between contiguous residues and evolutionary data [45]. While these methods have been successful in generating networks, testing whether an allosteric signal is transmitted in the pathway remains difficult to verify.

An especially successful and widely applied method for finding allosteric pathways has been put forth by Ranganathan and coworkers [39,46,47]. The statistical coupling analysis (SCA) method examines multiple sequence alignments for covariance, and subjects that to spectral decomposition to obtain the sectors. Networks have been identified in the RXR heterodimer protein, dihydrofolate reductase, and the PDZ domain. The accompanying experimental studies of the pathways have lent support to the proposed pathway signaling. Domain insertion scanning creates chimeras containing an insertion of an extra domain to perturb potentially allosteric sites. The 39 surface sites of the PDZ domain SCA network have been exhaustively mutated, and 11 showed significant effects on binding affinity for the peptide ligand. While these strategies have advanced the rigor of the predictions to validate the role of the residues, a means of unequivocally demonstrating that the residues participate in a contiguous signaling network and/or an energy network is difficult to deconvolve. Suggestions that contiguous pathways similar to those proposed by these methods may exist alongside energetic pathways [38,40] beg the question as to whether energetic pathways lacking the constraint of contiguity could exist and be missed by current lines of investigation.

We have drawn upon path finding, a well-known problem from the graph theory area of mathematics, to investigate the probability of paths in the model protein systems. A graph is a network consisting of nodes connected by edges to the nodes with which they interact [48,49]. In our work, nodes represent amino acid residues in the protein, and edges represent the residues within a specified cutoff distance as measured in three dimensional space using the xyz coordinates of the structure. The goal of the algorithm is to find the shortest path from the starting node to the end node in the fewest moves, preferably in a short amount of computational time. To determine the significance of the pathways in proteins, we are randomly selecting a subset of the residues in the protein and asking whether they make a contiguous pathway between the allosteric side and the ligand binding site. We are therefore making a graphical representation of a subset of the protein, and searching for the shortest path as evidence for the existence of any path between the allosteric and binding sites within the protein. If a shortest path exists then a contiguous pathway between the allosteric and binding sites exists within the protein in the chosen subset of residues. If a shortest path does not exist, then a contiguous pathway does not exist. We need not specifically find the shortest path. However, if any path, including the shortest, exists, the criterion for one existing pathway has been met, and therefore is a valid approach, allowing us to take advantage of the algorithm from graph theory.

Several algorithms can be employed to conduct path finding in a graph[50]. The simplest is the depth first search (DFS) [51–54]. The algorithm can be understood as follows. Start at the start position. For each node sharing an edge with the current node, check if it is connected to the end. If not, check if it shares edges with any additional nodes. If no, it is a dead end and therefore does not lead to a successful path; skip it. If yes, recursively continue the search. This algorithm is guaranteed to find a path if it exists, but as an exhaustive brute force search it is the least efficient method.

The breadth first search (BFS) algorithm [55] provides an alternative strategy with improvement on search time for a path. This algorithm guarantees a path to the goal (if it is possible and it guarantees finding the shortest one. The algorithm works by searching all the nodes immediately. The current one succeeding the current one before stepping deeper into the search. The other option are returned to the queue in case the shortest path is not found and these options need to be explored.

The A* algorithm [56–58] introduces a cost function as a means of including a priori knowledge to bias the search. Thus an intelligent next best step can be taken to maximize the chances of completing a path. The cost function f(x) is given as the sum of the functions g(x) and h(x). The function g(x) represents the cost to arrive at the current node. The function h(x) represents a heuristic best guess estimate at the cost to reach the final node from the current position. A reasonable estimate is the Euclidian distance between the node and the end, and is often used. Similar to BFS, this is computed for all of the hierarchically equivalent next steps. The one with the shortest distance is chosen for the next move. This is the most efficient algorithm because it searches with an informed bias. In light of the possibility that both energetic and positional pathways could exist or coexist in allosteric proteins, and the challenges with unequivocally demonstrating experimentally that a signal travels in a specific path, the aim of this work is to establish a benchmark for the statistical significance of finding contiguous pathways in a protein. Because the size of the amino acid residues varies considerably, a numerical approach using the structures of proteins has been taken. We explore a null model for the random selection of 20% of amino acids in the model proteins, commensurate with the number taken from SCA analysis. In this work we report on the benchmarks for three model systems: PDZ domain, full length p53 protein including two intrinsically disordered regions, and MutS, a DNA repair enzyme featuring allosteric signaling between the ATP hydrolysis site and the DNA binding site over one of the largest signaling distances characterized to date.

## 2. Results

The success rate of pathways in PDZ, p53, and MutS were assessed using the two algorithms, depth-first search (DFS)[48] and A*[57]. Implementation of the A* algorithm was motivated by DFS limitations to analyze large molecules. Using DFS and a distance cutoff of 6 Å, 1 trial with 10 selections took just short of 2 hours (6,926 seconds) to run. Using A* decreased runtimes by ~6 orders of magnitude, taking 704 seconds to run 3 trials of 100,000 selections each, a calculation that would have taken over six years with the original algorithm.

**Fig 1:**
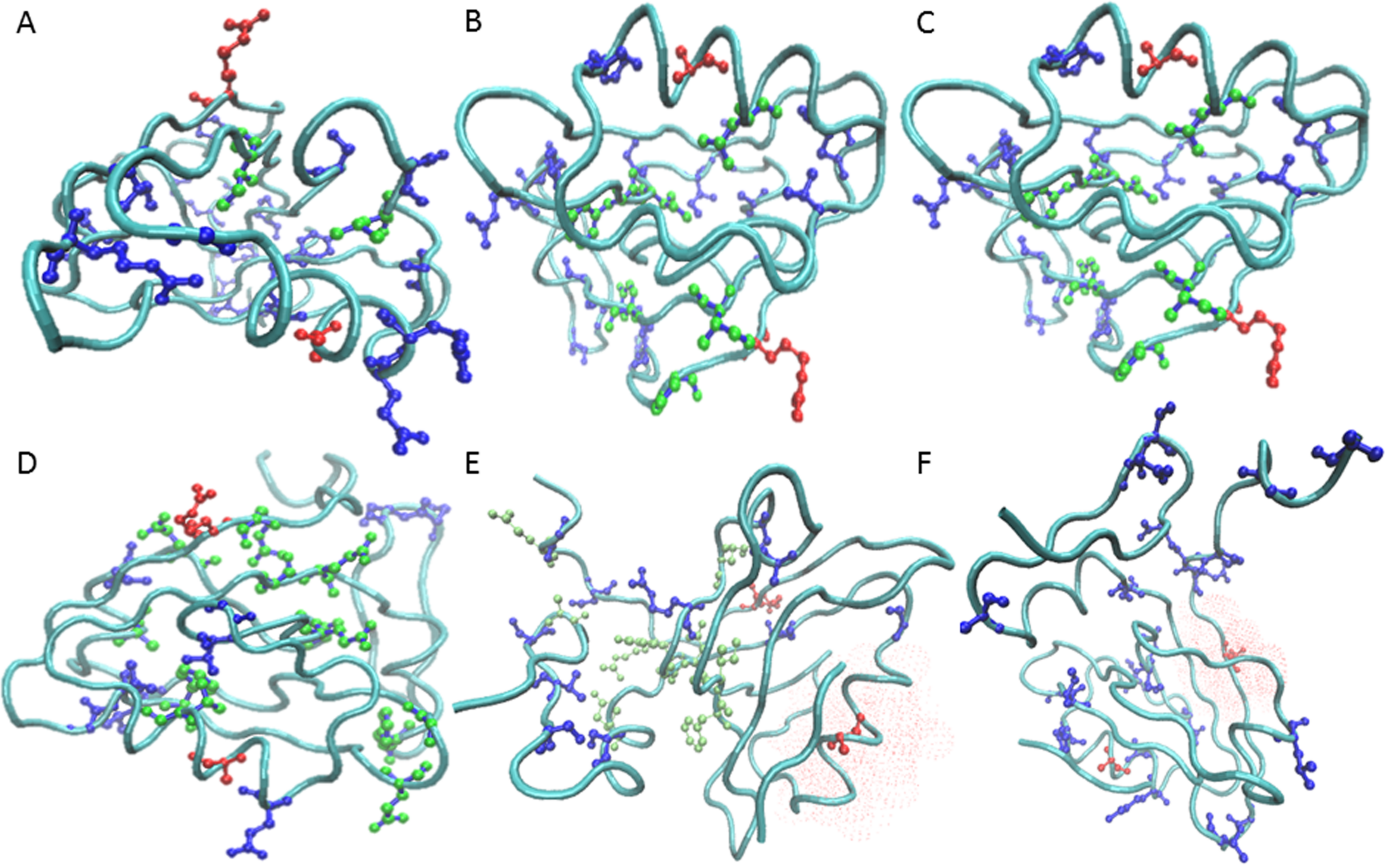
PDZ contiguous pathway examples. The PDZ protein backbone is shown in a cyan tube (PDB ID 1BE9). The allosteric signal travels between the allosteric effector binding site to the peptide ligand binding site (residues Arg 313 (top) and Ala 376 (bottom) highlighted in red ball and stick representation). The 20% of residues randomly selected for the trial appear in dark blue ball and stick. In panels (A-D), lime green balls highlight residues forming a successful contiguous pathway at the 6Å cutoff level. A short, direct path of 4 (A) and 5 (B) residues connects the sites. Longer meandering paths were also discovered: 8 residues (C) and 14 residues (D). In panels (E) and (F), examples of failed pathways are shown. A red surface shows where the pathway failed to connect.

**Fig 2:**
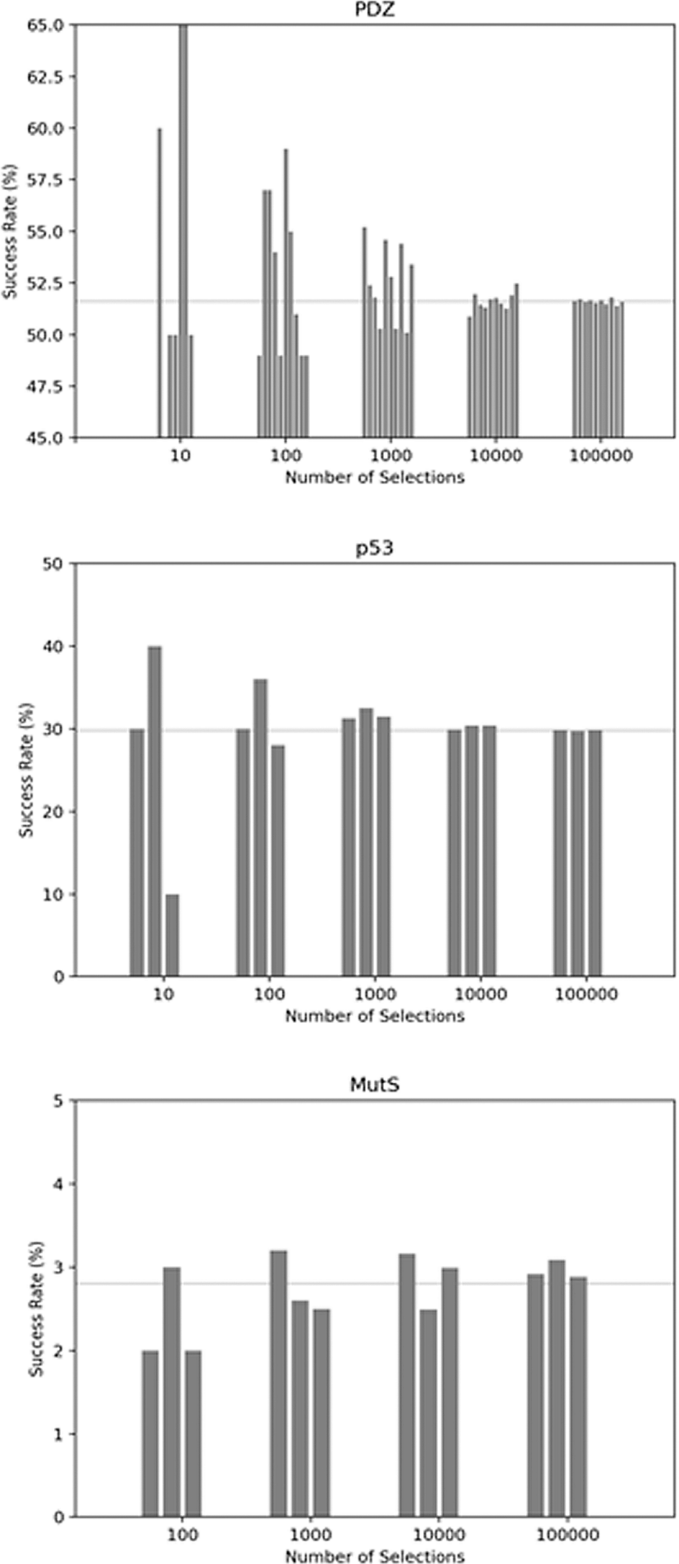
Pathway success rates. Convergence of the successful formation of contiguous pathways was determined for each model system. The x-axis indicates the number of selections of random residues. The rate at which any pathway connecting the active and allosteric site, subject to the 6Å distance constraint, is shown on the y-axis. The horizontal line indicates the converged value. (A) PDZ (number of trials = 10 at each selection level), (B) p53 (number of trials = 3 at each selection level), (C) MutS (number of trials = 3 at each selection level).

Our findings indicated that the prevalence of contiguous pathways depends on the system. In the model system PDZ, the rate at which successful pathways were found using 20% of the protein residues was 51% (Fig 1, Panel A). Fig 1 Illustrates some sample pathways found by the algorithm using the 6Å cutoff, as well as examples where the pathway failed to connect the active and allosteric site. For example, the pathway in Panel A progresses from residues 376 to 367 to 388 to 313, and the selection for that pathway was the set {308, 313, 316, 318, 327, 337, 348, 361, 366, 367, 370, 374, 373, 376, 383, 388, 394, 398, 401, 405, 410, 411, 415}. The length of the pathways varied from short and direct (4 residues, panel A) to long and meandering (12 residues, panel D). The algorithm identified lack of contiguous pathways for given selections due to failure to find selected neighbors around the start or end, or a lack of a nearest neighbor to complete any contiguous path between the ends. Fig 2 shows the rate of successfully obtaining pathways with a random selection of 20% of the residues in the protein. The number of selections was varied for each of the systems in order to determine the number of runs required to reach convergence. Since PDZ was the smallest system studied, it was ideally suited for carrying out pilot studies to gauge an appropriate number of selections and trials required to ascertain convergence on the success rate of pathway formation. Ten trials with the number of selections of protein residues varying from 10 to 100,000 were carried out (Fig 2A). With 10 selections per trial, the success rate was not yet converged, but the convergence around 51% was evident with 1,000 trials, which was confirmed by trials with 100,000 selections (51%, stdev = 0.12).

**Fig 3:**
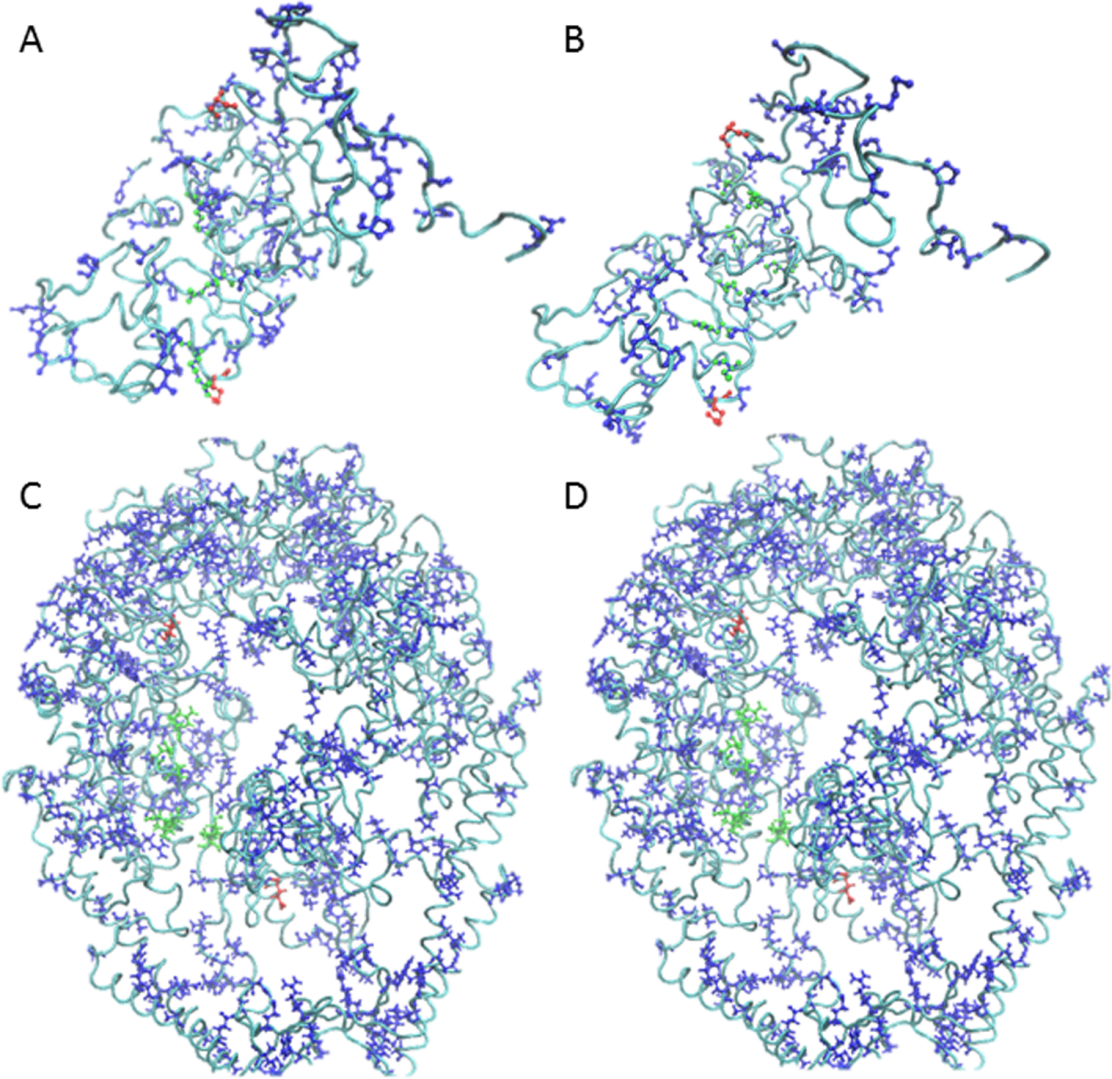
Successful pathway examples in p53 and MutS. The rendering and color scheme is as in Fig 1. (A,B) Contiguous p53 pathways connect the post -translational modification site Ser 46 (top red ball and stick) to Lys 120 (bottom red ball and stick) in the DNA binding domain at the interface with the DNA. (C,D). MutS pathways connect the ATP binding site residue Val 561 (top red ball and stick) with Leu 41 (bottom red ball and stick) interacting with DNA in the active site.

Although signaling pathways in p53 due to post translational modification have not yet been studied experimentally to our knowledge, the results indicate that connecting the site of modification with the DNA binding interface is possible. Fig 3 panels A and B illustrate two of the numerous possibilities. The success rate converged at 30% (stdev = 0.078, 1,000 selections) with a similar number of trials and selections as PDZ (Fig 2 Panel B). This was also confirmed by carrying out further trials out to 100,000 selections each. For the largest and structurally most complex protein, MutS, the success rate dropped to 3% (stdev = 0.11 at 1,000 selections, Fig 2 Panel C). The previously reported SCA pathway in MutS did meet the signaling criteria as expected. Thus, moving forward we opted to work at the 1,000 selection level as a reasonable compromise between computation time and convergence level for all three proteins.

**Fig 4:**
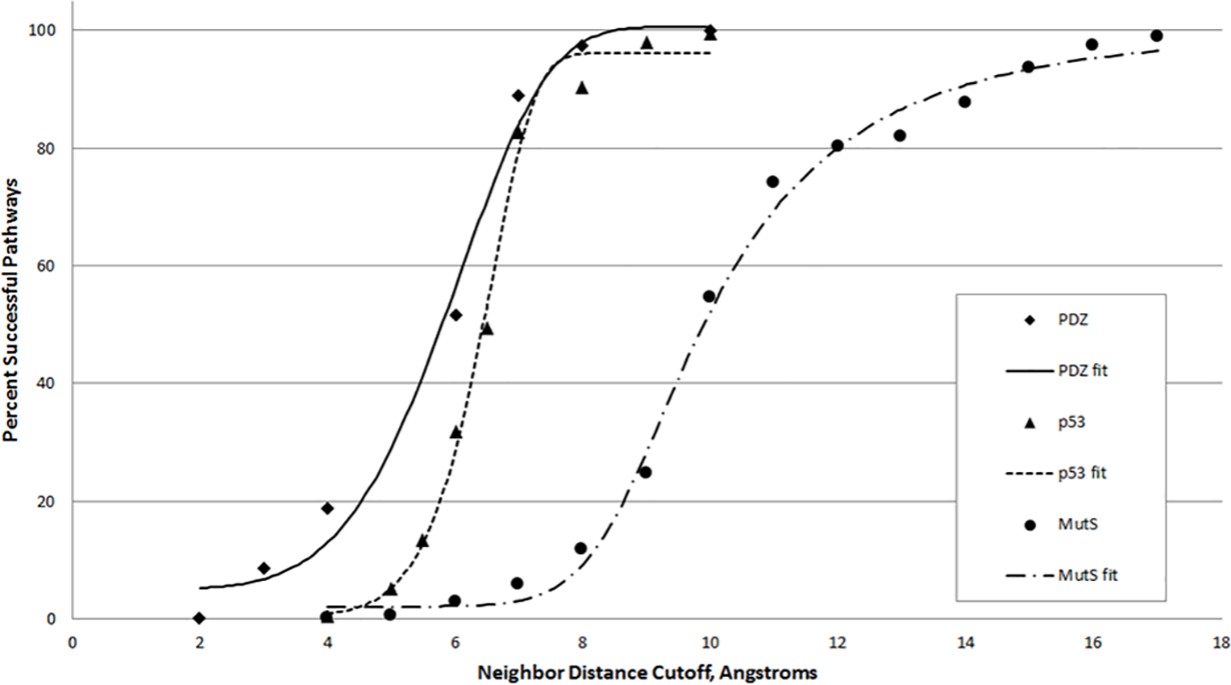
Success of pathways as a function of neighbor distance cutoff. The distance requirement for the contiguous pathway was varied (number of selections = 1000, number of trials = 3) for the three model systems, and was fit with a logistic curve. The best fit equations by system are given by system. PDZ: y = 100.663 + (5.015053–100.663)/(1 + (x/150.7424)^5.362259)^24925470 p53: y = 96.11989 + (0.4398672–96.11989)/(1 + (x/33.24754)^10.47436)^21720600 MutS: y = 100.1441 + (2.080954–100.1441)/(1 + (x/8.69924)^15.53203)^0.3183134.

To test the variation of the results based on the chosen 6Å cutoff, values encompassing 0 to 100% of the residues as neighbors was tested (Fig 4). All three curves follow a general sigmoidal shape and differ in the point at which they attain 95% pathway rate.

## 3. Discussion

To understand the likelihood of emergence of contiguous pathways and their uniqueness we have undertaken a study involving the random selection of 20% of amino acid residues in three proteins of various sizes and structural complexity. Allosteric signaling in proteins could occur via a contiguous pathway or through an energy landscape perturbation that does not necessitate the change of the ensemble average. Up to this point, studies of allosteric signaling via contiguous pathway signaling have been most prevalent in the literature, although the energy landscape model is not without precedence. The nature of the energy landscape signal renders it more difficult to detect experimentally, although it could exist either on its own or alongside a pathway mechanism. In this study we found contiguous pathways to emerge in all systems tested with a frequency ranging from rarely (3% in MutS) to about half of the trials (51% in PDZ).

Then range of success of pathways across the three model systems suggests that the significance of pathways may occur in a system specific manner. The pathways are most significant in the system exhibiting the greatest degree of complexity. MutS is a multidomain protein with 12 subunits, and also exhibits the greatest geometric complexity with its characteristic “theta” shape. The p53 protein also has multiple domains. Both of these systems may be susceptible to occurrence of bottlenecks in network connectivity due to geometrical constraints. A similar situation exists in p53, in which the pathway must extend across two domains in order to connect to the DNA binding site. Since N and C terminal domains are known to be highly mobile, connection as a bottleneck may become even more pronounced if the dynamic structure were to be taken into account. The single domain globular nature of PDZ lent itself to the most frequent emergence of pathways.

The pathways found in this study have revealed insight into the model systems studied and suggest a protocol to gain insight into other systems of interest. In PDZ, a pathway of contiguous residues has already been proposed by SCA and tested through mutagenesis of its surface residues [39]. Our studies suggest that pathways in globular proteins such as PDZ may exist with great abundance, since they appeared in more than half of the trials. Evolution may select for multiple signaling pathways simultaneously. Such redundancy would shore up the integrity of signaling in these proteins against mutational insults. Furthermore, many of these pathways may not be involved in signaling at all. Identifying residues involved in allosteric signal transduction constitutes an important first step, but the abundance of paths purely by chance underscores the importance of taking predictions as viable hypotheses for further testing. Still even with further testing, be it by computational or experimental means, proving which network was involved remains difficult. The possibility that false positives for signaling pathways in globular proteins may be especially high suggests that energetic based signaling, which does not require a contiguous network of residues, may also be playing a role. The existence of a contiguous pathway is not necessarily exclusive of other pathways.

The guardian of the genome, the p53 tumor suppressor protein has been considered here in its full length as a putative allosteric protein on the basis of reports of changes in DNA binding affinity due to distal post translational modification [59,60]. Adopting this view has allowed us to propose that contiguous signaling pathways in this protein could exist. The emergence of pathways in this protein appears less frequently than in the globular PDZ but more frequently than in the large multi-subunit MutS. Given that post translational modifications frequently occur in the disordered N and C terminal regions [61,62], signaling via a change in dynamics that is propagated through a contiguous or energetic network constitutes an interesting avenue to pursue.

The DNA repair enzyme MutS is the most complex protein in the study, and also the most difficult in which to find viable contiguous pathways. This lends itself to the idea that pathways, particularly those with verification, are likely to be more significant than those in simpler proteins. We were able to verify that the previously identified path is found by this method. The connectivity suggests that pathways in such complex proteins likely will involve overcoming bottlenecks, a probable obstacle for the emergence of contiguous signaling pathways in large proteins.

**Fig 5:**
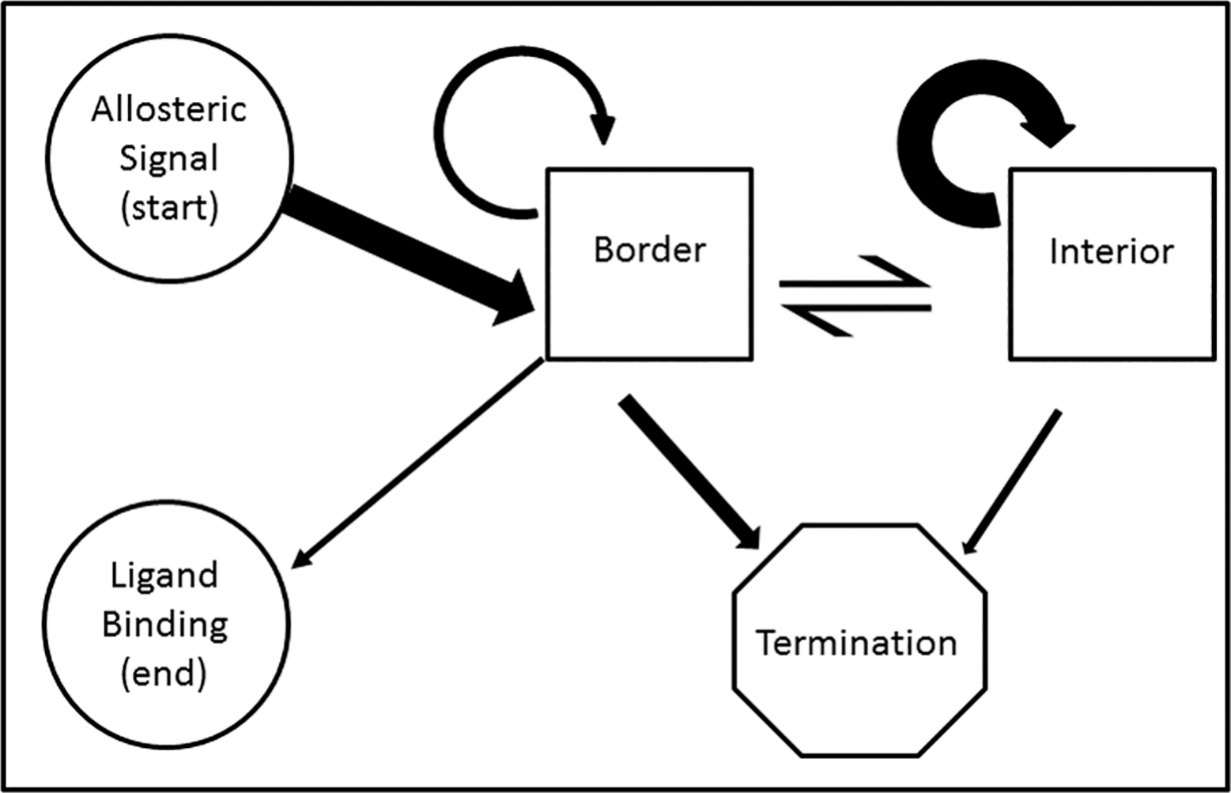
Contiguous pathway as a Markov process. Allosteric signaling via a contiguous pathway can be represented as a Markov process along a series of protein residues. The signal originates at the allosteric site and proceeds to the border state. This may transition to itself, or to the interior state. Either may transition to the termination state, indicating a pathway that failed to connect the allosteric site to the active site. The interior state differs from the border state in that residues in this state have a full complement of neighbors packed around them, whereas residues on the border lack some neighbors due to geometry. The thickness of the arrows schematically represents the frequency of transition between states. The self transition of the interior state tends to be higher than the self transition in the border state. Conversely, the border state tends to transition to the termination state more frequently than the interior state.

Geometric considerations in the formation of contiguous signaling pathways can be understood in terms of a Markov process (Fig 5). The path begins at the site of allosteric effector binding and terminates at the ligand binding site. A successful path iterates through transitions within or between the boundary and/or interior states until the end is reached. A failing pathway terminates through transitioning to the termination state representing no further residues within the neighborhood of any pathways can be found. The properties of the border and interior states reflect differences based on the number of neighbor residues in each category. In the interior state, residues have many neighbors whereas in the border state, the number of neighbors is lower. Having fewer neighbors decreases the chances of having a neighbor chosen in the selection to continue the path. Therefore, border states have a higher propensity to transition to the termination state.

Considering this model, the difference between pathway frequencies can be understood as a change in the equilibrium between the border and interior states. Because the number of neighbors a given protein has depends upon the local packing and size of its side chain as well as those in its vicinity, using a numerical solution to empirically observe the pathway frequency is required for each system of interest. To understand the general trends, a lattice protein model with systematic modulation of residues of regular size could be developed. However, to understand the trends from this study, let us assume that the densities of the packed portion of the proteins are equal and that the average number of neighbors does not change between proteins. PDZ, being a globular protein, has the smallest surface to volume ratio and therefore the least residues on the border. In this case, the model will be in the interior state most of the time, most residues will have ample neighbors such that the randomly chosen residues will frequently appear in the list of neighbors, and the next step is often possible. However, in the case of more geometrically complex proteins, the geometry dictates that the surface to volume ratio is larger; more states have fewer neighbors. Thus the path is more prone to termination. This is exemplified in p53 and to a greater extent in MutS, which both require multidomain signaling to transmit the signal. In the Markov model, this is represented by a higher probability to transition to the border state, which in turn is more likely than the interior state to transition to the termination state.

This idea is also borne out in the study of varying cutoff distances. When the cutoff approaches a bond length, pathways can not be formed. As the cutoff becomes larger, the larger the sphere encompassing neighbors becomes, and the more likely a residue from the random 20% selection will be a viable next step. For the smaller PDZ and p53 proteins, pathways were readily found when the cutoff was around 10Å. However, for the much larger protein MutS, a cutoff of around 18Å was required to observe the same effect.

The unexpectedly high prevalence of contiguous networks in light of the possible existence of energetic pathways which do not have the contiguity constraints opens the possibility that more energetic pathways than have been recognized up to this point may exist or coexist with contiguous pathways. The duplication of the signaling in proteins could be an evolutionary mechanism by which the essential functionality of signaling is preserved in a robust form resistant to point mutations.

The elucidation of all possible allosteric networks in proteins could bear importance on rational design of allosteric drugs. With the emergence of means by which protein networks can be predicted coupled with development of tests to verify allosteric control, engineering small molecules to interact with the sector is now becoming possible. Recognition of all possible networks could open greater opportunities for allosteric control of proteins through rational design strategies.

In conclusion, we have successfully tested the emergence of pathways in proteins by randomly selecting 20% of the amino acid residues and asking what percent of those trials connect points of allosteric regulation to the binding site in a continuous pathway. The findings indicate that the answer varies considerably with the system, but some general principles have emerged. Pathways most readily formed in the small globular protein and increasing size and complexity made their emergence more challenging. Thus we have provided benchmarks for the significance of randomly generated pathways, and suggest a method by which other systems of interest may be tested. We suggest that the observed trends may be considered as a Markov process, with the most important factor being the prevalence of neighbors to determine how readily paths may form. Given that pathways may be more abundant than previously thought particularly in globular proteins, the importance of verifying the signaling paths becomes apparent. Furthermore, it has suggested that several pathways may coexist. The existence of contiguous pathways does not necessarily rule out the possibility of other signaling pathways.

## 4. Methods

Protein model systems. The model systems chosen were PDZ, p53, and MutS, selected to span a range of sizes and shapes. PDZ is a well-known relatively small allosteric protein in which a pathway has already been posited [39]. The starting structure was the crystal structure 1BE9 [63]. Note that the numbering of residues as found in the crystal structure, 301 to 415, was maintained. MutS was chosen as a multidomain allosteric protein with a 100 Å distance traversed by the signal. The starting structure was the 1NNE crystal structure [64], with some minor adjustments made to convert it into the biologically relevant structure as previously reported [38,65]. The protein p53 was chosen for its intermediate size and complexity, having a post-translationally modified site at S46 in the N-terminal domain that propagates to affect DNA binding in the DNA binding domain [59,60]. Signaling pathways in p53 have not yet been reported to our knowledge, but may be of interest for further studies of the mechanism of action of this protein critical for cancer prevention. The starting structure was an engineered structure of the full length sequence, as this has not yet been available as a crystal structure due to the highly flexible N and C terminal domains. The UniProt[66] sequence ID P04637 was used as input to the phyre structure prediction [67] and the full length protein was obtained.

Casting the problem in graph theory. A graph G is defined G = (N,E,f(x)) in which each node n ∈ N represents a state, which in our case is an amino acid residue in a protein. Each edge is defined as the transition between nodes in Euclidean space or edge (n,n′) ∈ E. In our work, an edge is drawn if the Euclidean distance between amino acid residues is below a specified cutoff c. Search algorithms can be used to find paths within graphs. In the A* algorithm, f(x) is the heuristic cost function and is defined as the sum g(x) + h(x). g(x) represents the path cost function, indicating the cost from the start to the current node n_i_. h(x) is the heuristic estimate of the cost from node n_i_ to the end node n_end_. Here, to obtain the value for h(x) take the straight line Euclidean distance from the xyz coordinates of the amino acid at n_i_ to the amino acid at n_end_ as the distance of the residues using distances of residues in the pairwise distance matrix.

Pairwise distance matrix. The coordinates for the model systems were converted to the native crd and prmtop formats using tleap from the AMBER suite of programs [68–70]. In order to compute the distances between residues for establishing the specified cutoffs for viable pathways, the nativecontacts function from the AmberTools14 Suite [69,71] computed the pairwise atomic distances between residues and returned the minimum. This was used to generate a residue by all residue distance matrix. A python dictionary was constructed for each distance criterion for each model protein. A dictionary key was created for each residue in the protein, and its value corresponded to a list of the neighboring residues within the desired distance. This translated the molecule to a graph data structure.

**Fig 6:**
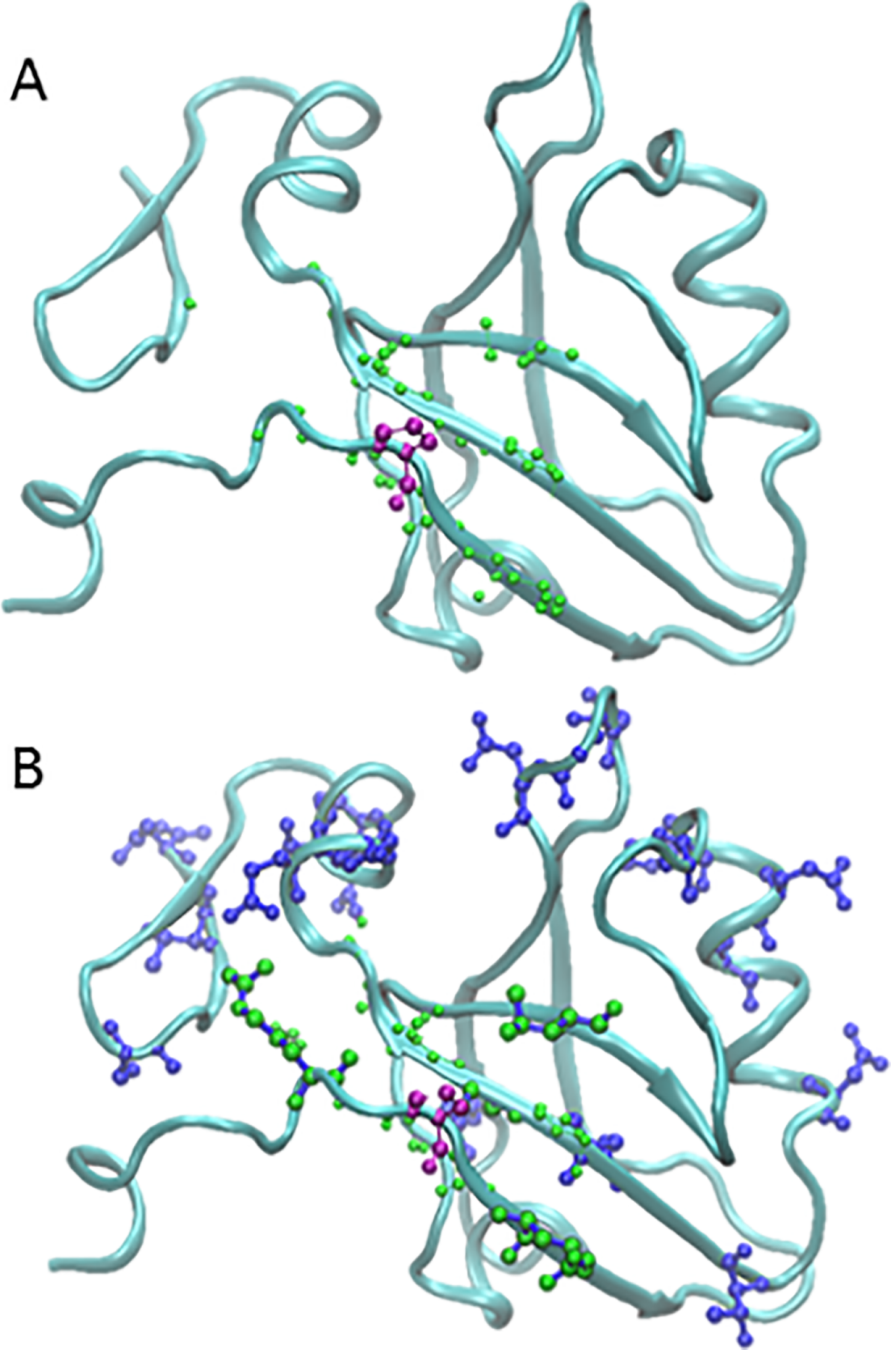
Selection process for finding pathways. (A). In purple is arbitrarily selected residue 311 of PDZ (1BE9)[63] surrounded by the residues within 6A (lime). (B) Overlaid on panel A are the randomly selected residues representing 20% of the protein (dark blue). The residues that are both within the 6Å cutoff and were included in the random selection have blue bonds and lime spheres. In this case, four residues (309, 313, 360, 390) met both criteria and are therefore possible next steps in the pathway.

Selection of subset of residues. To carry out the pathway search, for each trial 20% of the protein residues were selected at random. This percentage was chosen to be commensurate with the number of residues generated by the SCA pathway method [39,72,73]. In each combination, the starting point (allosteric site) and ending point (active site) were fixed, and the rest of the 20% were selected randomly. A reduced version of the original dictionary can be created containing only these selected points in both key and values, generating a smaller graph each time as a subset of the original. This guaranteed that pathways could only be made between residues that were both sufficiently close and included in the random 20% selection (Fig 6). The Python code used for this project is available in the Supporting Information (S1 File). The Visual Molecular Dynamics software [74,75] rendered all molecular visualizations.

Convergence. Since there are C(113, 21) (~ 10^20^) choices in PDZ and C(1528, 304) (~10^20^) choices in MutS, experimentation on the number of selections required us to determine convergence with use of a subset in order to make the calculations tractable. For a given selection of 20% of the residues, we evaluate whether a pathway can be found. For a given trial, a specified number of selections was chosen. We sequentially increased the number of selections per trial until batches of 10 trials were converged. Thus, the sampling was replicated at two independent levels. Once the number of required selections was determined, we then varied the distance criterion, the criterion for forming pathway between any two residues. As described above, separate corresponding dictionaries were used. The online program MyCurveFit from MyAssays Ltd. was used to fit 5 parameter logistic curves (asymmetric sigmoidal curve) to the data using the equation $y=d+\frac{a-d}{{\left[1+{\left(\frac{x}{c}\right)}^{b}\right]}^{m}}$ where a through d are fit parameters.

Path finding with search algorithms. We used a depth first search algorithm (DFS, described in Introduction) [48] to exhaustively search for pathways in PDZ to determine whether a pathway existed using the given selection. This method was chosen as a proof of concept to determine whether a pathway could exist by exhaustively checking every single one. Since our criteria for success is only whether a pathway can be created, some strategies were implemented to better use a traditional depth-first search algorithm. If the start or end points have no nearby points, the combination fails. Our DFS algorithm also chooses the option closest to the end point first. While still an exhaustive search, it finds the direct paths faster than an ordinary DFS. In an effort to run the much larger system MutS, a more tractable solution was required due to the computation time. We employed an A* search algorithm (described in the introduction) [57] and used the first as a point of validation on PDZ. The A* search evaluated each selection for whether a pathway existed. We increased the number of selections per trial until we saw convergence across trials. The Python 3.4 code was run on a MacBook Pro 2014 with 2.8GHz Intel Core i7 processor with 16G of ram, or on Wesleyan University’s High Performance Computing cluster, Microway GPU-HPC, parallelized across 2 K20 GPUs having 256 GB of memory.

## Supporting information

S1 File Python code used for this project to carry out the pathway searches is provided. (PY)
